# Oncological Feasibility of Conservative Axillary Surgery (Opinion Article): Tailored Axillary Surgery vs. Axillary Reverse Mapping-Guided Axillary Lymph Node Dissection

**DOI:** 10.3390/cancers18050854

**Published:** 2026-03-06

**Authors:** Masakuni Noguchi, Yusuke Haba, Emi Morioka, Masafumi Inokuchi

**Affiliations:** Department of Breast Surgery, Kanazawa Medical University Hospital, Daigaku 1-1, Uchinada, Kahoku 920-0293, Ishikawa, Japan; haba@kanazawa-med.ac.jp (Y.H.); emi-mori@kanazawa-med.ac.jp (E.M.); inokuchi@kanazawa-med.ac.jp (M.I.)

**Keywords:** axillary lymph node dissection, axillary reverse mapping, breast cancer, conservative axillary surgery, tailored axillary surgery

## Abstract

Background: Tailored axillary surgery (TAS) and axillary reverse mapping (ARM)-guided axillary lymph node dissection (ALND) have been developed to avoid arm lymphedema without increasing a risk of axillary recurrence. However, the oncological feasibility of TAS and ARM-guided ALND remains a crucial consideration. Methods: This article reviewed the oncological feasibility of TAS and ARM-guided ALND based on the current literature. Results: For ALND performed after TAS, additional involved nodes were found in 70% of upfront surgery patients and 60% of neoadjuvant chemotherapy (NAC) patients. ARM nodes were also involved in up to 64.7% of patients after ALND. However, it is not necessary to preserve all ARM nodes and lymphatics because multiple ARM lymphatic pathways exist. Selective preservation of ARM nodes closest to the axillary vein significantly reduced the incidence of involved ARM nodes (from 64.7% to 15.7%). Conclusions: TAS and ARM-guided ALND remain much less radical than ALND. However, residual nodal disease after TAS or ARM-guided ALND does not always develop axillary recurrence. Postoperative irradiation is effective in achieving local control in patients with low-volume (microscopic) residual nodal disease after TAS or ARM-guided ALND. We await the long-term results of prospective randomized clinical trials comparing TAS and ARM-guided ALND with conventional ALND.

## Simple Summary

Conventional axillary lymph node dissection (ALND) is associated with postoperative morbidities, including arm lymphedema, seroma, reduced shoulder mobility and local sensory dysfunction. Sentinel lymph node (SLN) biopsy has been introduced for assessing axillary nodal status in clinically node-negative (cN0) patients. However, ALND continues to be indicated for staging purposes and regional control in clinically node-positive (cN+) patients. Tailored axillary surgery (TAS) and axillary reverse mapping (ARM)-guided ALND have been developed to avoid arm lymphedema without increasing a risk of axillary recurrence. However, because TAS and ARM-guided ALND are much less radical than conventional ALND, their oncological feasibility remains a crucial consideration. For conventional ALND performed after TAS, additional involved nodes were found in 60–70% of patients. ARM nodes also were involved in 15.7–64.7% of patients who underwent conventional ALND. In this context, postoperative radiotherapy may be effective in preventing axillary recurrence in patients with low-volume (microscopic) additional nodal disease. And palpable suspicious axillary lymph nodes should be removed, not only in TAS but also in ARM-guided ALND. However, the majority of breast cancers are hormone receptor-positive and exhibit late recurrence. Therefore, we await the long-term results of prospective randomized clinical trials comparing TAS and ARM-guided ALND with conventional ALND to establish oncological safety of these procedures.

## 1. Introduction

Axillary surgery is a key component of breast cancer surgery for staging and treatment. However, ALND is postoperatively associated with several morbidities, including arm lymphedema, seroma, reduced shoulder mobility and local sensory dysfunction. Notably, the risk of arm lymphedema after ALND was reported to be 25% in a recent review [1]. This has often been used as an argument against ALND. Sentinel lymph node (SLN) biopsy has been performed for assessing axillary nodal status in clinically node-negative (cN0) patients [2,3]. Since its introduction, axillary surgery for breast cancer patients has changed tremendously over the past few decades. Currently, ALND can be avoided not only in cN0 patients with negative SLNs [4,5] but also in cN0 patients with fewer than three positive SLNs receiving breast radiation or axillary radiation [6,7]. Furthermore, ALND may be avoided in clinically node-positive (cN+) patients whose nodal status becomes SLN-negative following neoadjuvant chemotherapy (NAC) [8]. However, ALND continues to be indicated for staging purposes and for the treatment of regional disease in a significant number of cN+ patients [9,10]. Recently, various forms of conservative axillary surgery such as TAS and ARM-guided ALND have been developed to replace or supplement conventional ALND. In the era of effective multimodality therapy, completion ALND removing all microscopic axillary disease may be not necessary in both cN0 patients and cN+ patients. However, there remains a lack of consensus among surgeons regarding conservative axillary surgery. Therefore, in this article, we evaluated the oncological feasibility of conservative axillary surgery including TAS and ARM-guided ALND.

## 2. Conservative Axillary Surgery

Conservative axillary surgery has been developed to avoid arm lymphedema without increasing a risk of axillary recurrence. It includes conservative axillary regional excision (CARE) [11], tailored axillary surgery (TAS) [12,13], partial lower ALND [14] and ARM-guided ALND [15,16] (Table 1). Conservative axillary surgery is conceptually divided into two groups. The first group includes TAS and CARE, which commonly remove SLNs and other palpable suspicious nodes, although TAS may optionally be performed with image-guided localization of the histologically involved clipped node. The second group includes ARM-guided ALND and partial lower ALND, which aim to preserve upper-extremity lymphatic drainage. Partial lower ALND preserves the axillary lymph nodes and lymphatics located between the second intercostobrachial nerve and axillary vein [14], whereas ARM-guided ALND is performed in a personalized fashion to preserve the lymphatics of the upper extremity [17,18]. In this article, we evaluate the oncological feasibility of TAS and ARM-guided ALND.

## 3. Tailored Axillary Surgery (TAS)

**Figure 1:**
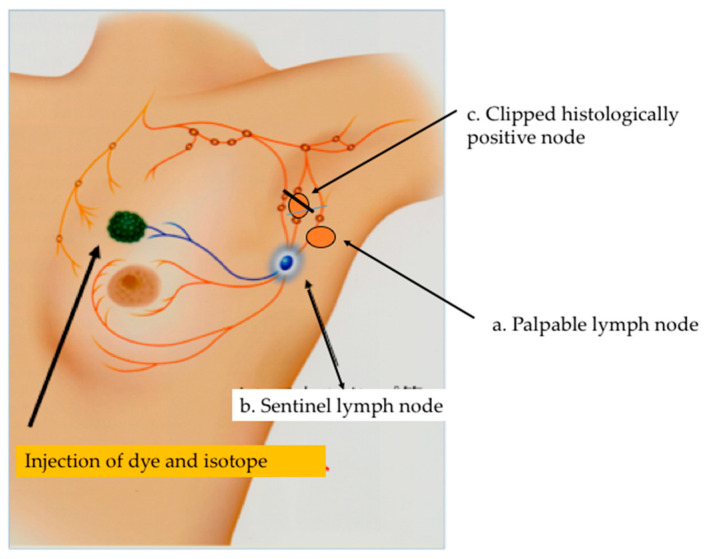
Tailored axillary surgery (TAS) removes all palpable suspicious lymph nodes together with the SLNs and clipped nodes to achieve optimal results. Residual, non-palpable nodal disease after TAS can be controlled by postoperative irradiation.

TAS was developed as an alternative to ALND for cN+ patients [12]. This procedure removes all palpable suspicious lymph nodes together with SLNs, and may be optionally performed with image-guided localization of the clipped node to achieve optimal results. TAS aims to convert a cN+ axilla into a cN0 one, mainly by palpation-guided selective removal of grossly involved nodal disease [19] (Figure 1). Postoperative radiotherapy is routinely used to achieve local control in patients with low-volume remaining nodal disease. However, the value of this procedure with respect to long-term regional control and survival needs to be investigated in a prospective randomized trial.

A randomized clinical TAXIS trial comparing TAS in combination with axillary irradiation to conventional ALND has been performed in NAC and upfront surgery settings. Although the selective removal of clipped nodes by image-guided localization is not a mandatory part of TAS, clipped nodes were removed in 94.3% of patients. All patients either undergo adjuvant whole-breast irradiation after breast-conserving surgery (BCS) or chest-wall irradiation after mastectomy. The ongoing TAXIS trial will determine whether axillary treatment with TAS and radiotherapy is oncologically non-inferior and associated with improved quality of life compared to conventional ALND in cN+ patients [12]. A total of 1500 patients was randomized and accrual completion was expected in 2025. An analysis of the primary endpoint will be published in 2030 [13,19].

However, for ALND performed after TAS in a phase II TAXIS trial, additional involved nodes were found in 70% of upfront surgery patients and 60% of NAC patients [12]. Although all these patients received postoperative irradiation after TAS, the incidence of additional involved nodes was much higher compared with corresponding rates from the Z0011 and AMAROS trials [6,7]. The incidence of additional involved nodes after SLN biopsy was 27.4% in the Z0011 trial and 33% in the AMAROS trial, in which most patients in the SLN biopsy-alone group did not develop axillary recurrence [20] (Table 2). Nevertheless, these trials are not directly comparable because they involved fundamentally different patient populations (cN+ in the TAXIS trial vs. cN0 in the Z0011 and AMAROS trials).

The landmark NSABP B-04 trial serves as a reference with relevant data. In its observation arm (no ALND or axillary radiation), ipsilateral axillary nodes were the first site of failure in only 18% of cN0 patients, despite an expected histological nodal positivity rate of 40% in cN0 patients. The rate of axillary recurrence was reduced to 3% after axillary radiotherapy in cN0 patients. On the other hand, the rate of axillary recurrence was reduced to 12% after radiotherapy in cN+ patients, despite an expected histological nodal positivity rate of 73% [21] (Table 2). When a cN+ axilla becomes cN0 after TAS, postoperative radiotherapy would reduce the axillary recurrence rate. It has been suggested that outcomes will be favorable in the TAXIS trial [12].

The TAXIS phase III trial is being conducted to test the hypothesis that TAS with axillary radiotherapy is non-inferior to conventional ALND in terms of disease-free survival of cN+ patients at first presentation in the era of effective systemic therapy and regional nodal irradiation [22]. We await the results of the TAXIS trial involving a large number of cN+ patients as they will help determine the benefit of TAS. Oncological non-inferiority is hypothetical until mature results from TAXIS phase III are available. Moreover, tumor biology should be considered when omitting ALND [23]. The biological and systemic therapy landscape has evolved significantly in the current cN+ population.

## 4. Axillary Reverse Mapping (ARM)–Guided ALND

**Figure 2:**
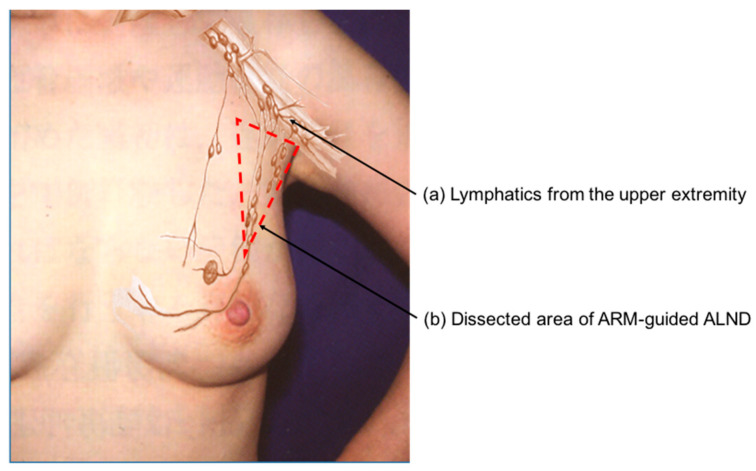
ARM-guided ALND: (a) ARM nodes and lymphatics in contact with the axillary vein are selectively preserved to pre-vent arm lymphedema; (b) A broken line indicates a dissected area of ARM-guided ALND.

The occurrence of arm lymphedema is mainly due to the unnecessary sacrifice of upper extremity lymphatics. ARM-guided ALND was developed to delineate and preserve the lymphatic drainage pathways of the arm during ALND, thereby minimizing arm lymphedema (Figure 2) [15,16]. The ARM technique is based on the hypothesis that arm lymphatics are distinct from breast lymphatics, and thus axillary nodes and lymphatics from the upper extremity are theoretically not involved. The ARM approach has the potential to reduce the incidence of arm lymphedema [24,25]. A meta-analysis including 1696 patients favored ALND with ARM compared with conventional ALND alone for preventing arm lymphedema occurrence (4.8% vs. 18.8% respectively; *p* < 0.0001) [26]. However, there have been concerns about the oncological feasibility of ARM due to the involvement of ARM nodes [24,25,27,28,29,30]. Anatomically, there are lymphatic interconnections between nodes draining from the upper extremity and nodes draining from the breast [31,32].

The ARM procedure was originally performed using blue dye [15,16]. A meta-analysis of studies using blue dye alone to identify ARM nodes reported ARM node and/or lymphatics identification rates ranging from 47% to 89% and metastatic involvement rates for ARM nodes ranging from 0% to 27% [33]. In contrast, when indocyanine green (ICG) fluorescence was used, identification rates of ARM node and/or lymphatics were found to range from 81% to 96%, with metastatic involvement rates for ARM nodes ranging from 3% to 22% [33]. Thus, fluorescence provides better intraoperative visualization of lymphatic vessels and nodes than blue dye [34,35,36,37,38].

**Figure 3:**
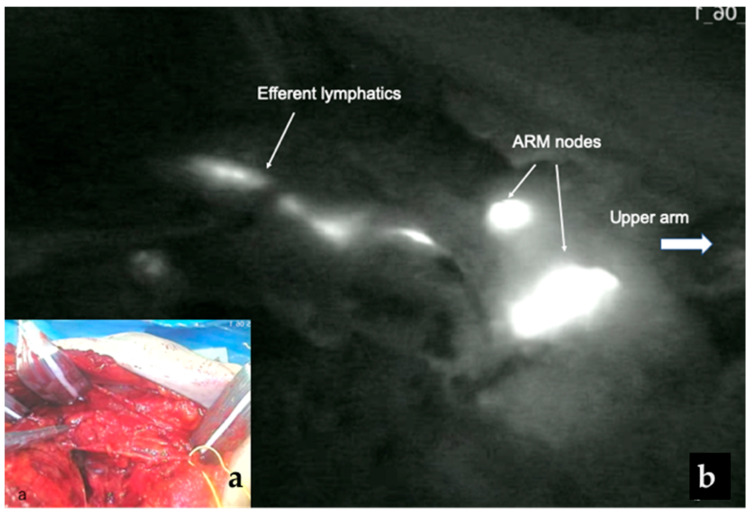
(**a**) Surgical field after ARM-guided ALND; (**b**) Fluorescent ARM nodes and lymphatics are visible around the axillary vein after ARNM-guided ALND.

The FARM trial was recently conducted to evaluate the utility of fluorescence ARM-guided ALND in breast cancer. In this study, fluorescent ARM nodes were successfully found and removed within the ALND field in 95% of patients (95/100) and were involved with metastases in 18.9% of patients. The rate of involved ARM nodes was not significantly different between the upfront surgery patients and the NAC patients [39] (Table 3). In our previous study using ICG fluorescence-guided ARM, however, fluorescent ARM nodes were involved in 35.4% of NAC patients and 64.7% of upfront surgery patients [40]. Because the fluorescence method is highly sensitive in visualizing ARM nodes and lymphatics, it may identify a greater number of involved ARM nodes. These finding suggest that lymphatics from the ARM are not entirely independent. Prolonged diffusion times may cause the ICG to spread to additional axillary lymph nodes, which might increase the identification of involved ARM nodes [41]. Nevertheless, it is not necessary to preserve all ARM nodes because ARM lymph nodes and lymphatics are multiple. Ortega-Exposito et al. [41] divided patients into two cohort based on ICG migration patterns: standard technique (removal of all ARM nodes) and targeted technique (selective preservation of ARM nodes in contact with the axillary vein). With selective preservation of ARM nodes closest to the axillary vein (targeted technique), the incidence of involved ARM nodes and lymphatics can be significantly reduced from 47.1% with the standard technique to 15.7% with the targeted technique (*p* = 0.026) [41] (Table 3). Thus, it is important to preserve ARM nodes around the axillary vein to prevent arm lymphedema (Figure 3).

Several randomized clinical trials have been conducted to compare conventional ALND and ARM-guided ALND [24,25,27,28,29,30]. Yuan et al. [24] developed a modified ARM approach called iDEntification and Preservation of ARm lymphaTic system (DEPART). In this DEPART study, fluorescent dye (ICG) and methylene blue were used to identify the ARM nodes and lymphatics. A total of 874 cN+ patients and 480 SLN-positive patients were randomized into two groups: 648 patients underwent conventional ALND and 543 patients underwent ARM-guided ALND with preservation of ARM nodes and lymphatics. In the ARM-guided ALND group, ARM nodes and lymphatics were preserved unless they coincided with SLNs and palpable suspicious nodes. Palpable suspicious ARM nodes were histologically examined by partial frozen sections in 59 patients and involved ARM nodes were removed in 38 patients (38/558, 6.8%). More than 60% of tumors were hormone receptor-positive in either group. After surgery, regional nodal radiation was administered to 60.6% of the conventional ALND group and to 56.5% of the ARM-guided group, and patients in both groups received adjuvant chemotherapy. Within a median follow-up period of 37 months, arm lymphedema was observed in 99 patients (15.3%) in the conventional ALND group and in 18 patients (3.3%) from the ARM-guided ALND group (*p* < 0.001), but the incidence of axillary recurrence did not differ significantly between the two groups (1.4% vs. 1.2%) [24].

Given these findings, intraoperative nodal palpation must be regarded as an essential component rather than an optimal adjunct in ARM-guided ALND [42,43,44,45]. Moreover, postoperative radiotherapy is necessary for preventing axillary recurrence in high-risk patients after ARM-guided ALND. If it is not possible to spare fluorescent ARM lymphatics, these patients could be treated with lympho-lymphatic or lympho-venous anastomosis at the time of surgery to reduce a risk of arm lymphedema [46,47]. However, the follow-up duration was insufficient to evaluate axillary recurrence after ARM-guided ALND. Since the majority of breast cancer is hormone receptor-positive, which recurs over a longer period of time [48,49], a longer follow-up is needed to confirm the oncological feasibility of ARM-guided ALND.

## 5. Conclusions

TAS and ARM-guided ALND have been developed to reduce the incidence of arm lymphedema without increasing a risk of axillary recurrence. However, there have been concerns about the oncological feasibility of these procedures. TAS and ARM-guided ALND remained much less radical than conventional ALND. Nevertheless, postoperative radiotherapy may be effective for preventing axillary recurrence in patients with low-volume additional nodal disease. If palpably suspicious axillary lymph nodes are found during surgery, therefore, these nodes should be removed not only in TAS but also in ARM-guided ALND. However, since conservative axillary surgery lacks prospective studies with sufficient follow-up periods, it should be considered an investigational approach. Ultimately, we await the long-term results of prospective randomized clinical trials comparing TAS and ARM-guided ALND with conventional ALND to establish the oncological feasibility of these procedures. Ideally, direct comparative studies between TAS and ARM-guided ALND would be of great interest.

## Figures and Tables

**Table 1:** Category of Conservative Axillary Surgery.

**a. Selective removal of SLNs and other palpable suspicious nodes**
TAS
CARE
**b. Preservation of the lymphatic drainage from upper extremity**
ARM-guided ALND
Lower partial dissection

ALND: axillary lymph node dissection; ARM: axillary reverse mapping; CARE: conservative axillary regional excision; SLN: sentinel lymph node; TAS: tailored axillary surgery.

**Table 2:** Trials of Axillary Dissection and Sentinel Lymph Node Biopsy.

Trials/ Reference Number	No. of Patients	Clinical Nodal Status	Axillary Treatment	% of Positive Nodes at ALND	% of Axillary Recurrence
ACOSOG Z0011 [6]	446	cN0	SLN biopsy with RT	27.4%	1.50%
AMAROS [7]	681	cN0	SLN biopsy with RT	33%	1.19%
NSABP B04 [21]	365	cN0	None	40%	18%
	352	cN0	RT alone	40%	3%
	294	cN+	RT alone	73%	12%

ALND—axillary lymph node dissection; SLN—sentinel lymph node; cN0—clinically negative node; cN+—clinically positive node; RT—radiotherapy; [ ]—reference number.

**Table 3:** Fluorescence-guided Axillary Reverse Mapping.

Authors/ Reference Number	No. of Patients	Nodal Status	Mapping Tracer	Identification Rate of ARM Nodes	% of ARM Nodes Metastases
Krishna et al. [35]	78	cN+ or cN0	Fluorescence	91%	9.0%
Nguyen et al. [39]	100	cN+ or SLN-positive	Fluorescence	95%	18.9%
Noguchi et al. [40]	90	SLN-positive	Fluorescence	Not reported	12.2%
	65	cN+ (NAC)	Fluorescence	Not reported	35.4%
	68	cN+ (upfront surgery)	Fluorescence	Not reported	64.7%
Ortega et al. [41]	41	cN+ or SLN-positive	Fluorescence	87.9%	15.7% (targeted technique)
		(upfront surgery or NAC)			47.1% (standard technique)

ARM—axillary reverse mapping; cN+—clinically node-positive; cN0—clinically node-negative; NAC—neoadjuvant chemotherapy; SLN—sentinel lymph node.

## References

[1] Bakri N.A.C., Kwasnicki R.M., Khan N., Ghandour O., Lee A., Grant Y., Dawidziuk A., Darzi A., Ashrafian H., Leff D.R. (2023). Impact of axillary lymph node dissection and sentinel lymph node biopsy on upper limb morbidity in breast cancer patients. Systemic review and meta-analysis. Ann. Surg..

[2] Krag D.N., Weaver D.L., Alex L.C., Fairbank J.T. (1993). Surgical resection and radiolocalization of the sentinel lymph node in breast cancer using a gamma probe. Surg. Oncol..

[3] Giuliano A.E., Kirgan D.M., Guenther J.M., Morton D.L. (1994). Lymphatic mapping and sentinel lymphadenectomy for breast cancer. Ann. Surg..

[4] Krag D.N., Anderson S.J., Julian T.B., Brown A.M., Harlow S.P., Costantino J.P., Ashikaga T., Weaver D., Mamounas E.P., Jalove L.M. (2010). Sentinel –lymph-node resection compared with conventional axillary-lymph-node resection in clinically node-negative patients with breast cancer: Overall survival findings from the NSABP B-32 randomised phase 3 trial. Lancet Oncol..

[5] Veronesi U., Viale G., Paganelli G., Zurrida S., Luini A., Galimberti V., Veronesi P., Intra M., Maisonneuve P., Zucca F. (2010). Sentinel lymph node biopsy in breast cancer. Ten-year results of a randomized controlled study. Ann. Surg..

[6] Giuliano A.E., McCall L., Beitsch P., Whitworth P.W., Blumencranz P., Leitch A.M., Saha S., Hunt K.K., Morrow M., Ballman K. (2010). Locoregional recurrence after sentinel lymph node dissection with or without axillary dissection in patients with sentinel lymph node metastases. The American College of Surgeons Oncology Group Z0011 randomized trial. Ann. Surg..

[7] Donker M., van Tiernhoven G., Straver M.E., Meijnen P., van de Velde C.J.H., Mensel R.E., Cataliotti L., Westenberg A.H., Klinkenbijl J.H.G., Orzalesi L. (2014). Radiotherapy or surgery of the axilla after a positive sentinel node in breast cancer (EORTC 10981-22023 AMAROS): A randomized, multicenter open-label, phase 3 non-inferiority trial. Lancet Oncol..

[8] Mamtani A., Barrio A.V., King T.A., Van Zee K.J., Plitas G., Pilewskie M., El-Tamer M., Gemignani M.L., Heerdt A.S., Sclafani L.M. (2016). How often does neoadjuvant chemotherapy avoid axillary dissection in patients with histologically confirmed nodal metastases: Results of a prospective study. Ann. Surg. Oncol..

[9] Weber W.P., Hansen S.E., Wong D.E., Heidinger M., Montagna G., Cafferty F.H., Kirby A.M., Coles C.E. (2024). Personalizing locoregional therapy in patients with breast cancer in 2024: Tailoring axillary surgery, escalating lymphatic surgery, and implementing evidence-based hypofractionated radiotherapy. Am. Soc. Clin. Oncol. Educ. Book.

[10] Heidinger M., Weber W.P. (2024). Axillary surgery for breast cancer in 2024. Cancers.

[11] Cowher M.S., Gobmyer S.R., Lyons J., O’Rourke C., Baynes D., Crowe J.P. (2014). Conservative axillary surgery in breast cancer patients undergoing mastectomy: Long-term results. J. Am. Coll. Surg..

[12] Weber W.P., Matrai Z., Hayoz S., Tausch C., Henke G., Zwahlen D.R., Gruber G., Zimmermann F., Seiler S., Maddox C. (2021). Tailored axillary surgery in patients with clinically node-positive breast cancer: Pre-planned feasibility substudy of TAXIS (OPBC-03, SAKK 23/16, IBCSG 57-18, ABCSG-53, GBG 101). Breast.

[13] Maggi N., Nussbaumer R., Holzer L., Weber W.P. (2022). Axillary surgery in node-positive breast cancer. Breast.

[14] Kodama H., Mise K., Kan N. (2012). Partial lower axillary dissection for patients with clinically node-negative breast cancer. J. Int. Med. Res..

[15] Thompson M., Korourian S., Henry-Tillman R., Adkin L., Mumford S., Westbrook K.C., Klimberg V.L. (2007). Axillary reverse mapping (ARM): A new concept to identify and enhance lymphatic preservation. Ann. Surg. Oncol..

[16] Nos C., Lesieur B., Clough K.B., Lecuru F. (2007). Blue dye injection in the arm in order to conserve the lymphatic drainage of the arm in breast cancer patients requiring an axillary dissection. Ann. Surg. Oncol..

[17] Noguchi M., Inokuchi M., Yokoi-Noguchi M., Morioka E., Haba Y. (2023). Conservative axillary surgery is emerging in the surgical management of breast cancer. Breast Cancer.

[18] Noguchi M., Inokuchi M., Yokoi-Noguchi M., Morioka E., Haba Y. (2023). Conservative axillary surgery may prevent arm lymphedema without increasing axillary recurrence in the surgical managing of breast cancer. Cancers.

[19] Heidinger M., Knaauer M., Tausch C., Weber W.P. (2023). Tailored axillary surgery—A novel concept for clinically node positive breast cancer. Breast.

[20] Morrow M. (2013). It is not always necessary to do axillary dissection for T1 and T2 breast cancer. Cancer Res..

[21] Fisher B., Redmond C., Fisher E.R., Bauer M., Wolmark N., Wickerham L., Deutsch M., Montague E., Margolese R., Foster R. (1985). Ten-year results of a randomized clinical trial comparing radical mastectomy and total mastectomy with or without radiation. N. Engl. J. Med..

[22] Henke G., Knauer M., Ribi K., Hayoz S., Gerard M.-A., Ruhstaller T., Zwahlen D.R., Muenst S., Ackerknecht M., Hawle H. (2018). Tailored axillary surgery with or without axillary lymph node dissection followed by radiotherapy in patients with clinically node-positive breast cancer (TAXIS): Study protocol for a multicenter, randomized phase-III trial. BMC.

[23] Montagna G., Alvarado M., Myers S., Mrdutt M.M., Sun S.X., Sevilimedu V., Barrio A., van den Bruele A.B., Boughey J.C., Boyle M.K. (2026). Oncoplastic outcomes with and without axillary lymph node dissection in patients with residual micrometastases after neoadjuvant chemotherapy (OPBC-07/microNAC): An international, retrospective cohort study. Lancet Oncol..

[24] Yuan Q., Wu G., Xiao S.-Y., Hou J., Ren Y., Wang H., Wang K., Zhang D. (2019). Identification and preservation of arm lymphatic system in axillary dissection for breast cancer to reduce arm lymphedema events: A randomized clinical trial. Ann. Surg. Oncol..

[25] Yue Y., Zhuang D., Zhou P., Zheng L., Fan Z., Zhu J., Hou L., Yu F., Dong X., Xia L. (2015). A prospective study to assess the feasibility of axillary reverse mapping and evaluate its effect on preventing lymphedema in breast cancer patients. Clin. Breast Cancer.

[26] Co M., Lam L., Suen D., Kwong A. (2023). Axillary reverse mapping in the prevention of lymphoedema: A systemic review and pooled analysis. Clin. Breast Cancer.

[27] Beek M.A., Gobardhan P., Klompenhouwer E.C., Menke-Pluijmers M.B., Steenvoorde P., Merkus J.W., Rutten H.J.T., Voogd A.C., Luiten E.J.T. (2020). A patient- and assessor-blinded randomized controlled trial of axillary reverse mapping (ARM) in patients with early breast cancer. Eur. J. Surg. Oncol..

[28] Faisal M., Sayed M.G., Antonious K., Bakr A.A., Farag S.H. (2019). Prevention of lymphedema via axillary reverse mapping for arm lymph-node prevention following breast cancer surgery: A randomized controlled trial. Patient Saf. Surg..

[29] Abdelhamid M.L., Bari A.A., Farid M., Nour H. (2020). Evaluation of axillary reverse mapping (ARM) in clinically axillary node negative breast cancer patients –Randomized controlled trial. Int. J. Surg..

[30] Gennaro M., Maccauro M., Mariani L., Listorti C., Sigari C., De Vivo A., Chisari M., Maugeri I., Lorenzoni A., Alberti G. (2022). Occurrence of breast –cancer-related lymphedema after reverse lymphatic mapping and selective axillary versus standard surgical treatment of axilla: A two-arm randomized clinical trial. Cancer.

[31] Suami H., Taylor G.I., Pan W.-R. (2007). The lymphatic territories of the upper limb: Anatomical study and clinical implications. Plast. Reconstr. Surg..

[32] Pavlista D., Eliska O. (2012). Analysis of direct oil contrast lymphography of upper limb lymphatics traversing the axilla –a lesson from the past contribution to the concept of axillary reverse mapping. Eur. J. Surg. Oncol..

[33] Wijaya W.A., Peng J., He Y., Chen J., Cen Y. (2020). Clinical application of axillary reverse mapping in patients with breast cancer: A systemic review and meta-analysis. Breast.

[34] Noguchi M., Yokoi M., Nakano Y. (2010). Axillary reverse mapping with indocyanine fluorescence imaging in patients with breast cancer. J. Surg. Oncol..

[35] Krishna O.R., Arun H.N., Prakash B.V., Aparna S.M., Srinivas C., Altaf S., Akshatha G.T. (2025). Prospective study of axillary reverse mapping using indocyanine green in breast cancer. SSR Inst. Int. J. Life Sci..

[36] Foster D., Choy N., Porter C., Ahmed S., Wapnir I. (2018). Axillary reverse mapping with indocyanine green or isosulfan blue demonstrate similar crossover rates to radiotracer identified sentinel nodes. J. Surg. Oncol..

[37] Jupitz S.A., Lin C., Kawahara T., McKinney G., Uselmann A.J., Neuman H.B. (2025). Higher rates of visualization for axillary reverse mapping using indocyanine green fluorescence compared with blue dye. J. Surg. Res..

[38] Sakurai T., Endo M., Shimizu K., Yoshimizu N., Nakajima K., Nosaka K., Dai Y., Iwao A., Jinnai Y. (2014). Axillary reverse mapping using fluorescence imaging is useful for identifying the risk group of postoperative lymphoedema in breast cancer patients undergoing sentinel node biopsies. J. Surg. Oncol..

[39] Nguyen C.L., Poels D., Teoh B., Rastogi P., Seah J.L., Chan B., Graham S., Azimi F., Mak C., Pulitano C. (2025). Indocyanine green fluorescence-guided axillary reverse mapping for axillary lymph node dissection in breast cancer: The FARM trial. Ann. Surg. Oncol..

[40] Noguchi M., Inokuchi M., Yokoi-Noguchi M., Morioka E. (2023). The involvement of axillary reverse mapping nodes in patients with node-positive breast cancer. Eur. J. Surg. Oncol..

[41] Ortega-Exposito C., Pla M., Campos M., Falo C., Perez-Montero H., Azcarate J., Benitez A., Salinas S., Bosch J., Aranguena-Penacoba M. (2024). Axillary reverse mapping using indocyanine green in breast cancer: Standardization of the technique. Clin. Breast Cancer.

[42] Ikeda K., Ogawa Y., Komatsu H., Mori Y., Ishikawa A., Nakajima T., Oohira G., Tokunaga S., Fukushima H., Inoue T. (2012). Evaluation of the metastatic status of lymph nodes identified using axillary reverse mapping in breast cancer patients. World J. Surg. Oncol..

[43] Wu J.-D., Wang Z., Zeng H.-C., He L.-F., Zhang Y.-Q., Huang G.-S., Zhang F., Wei X.-L., Huang W.-H., Zhang G.-J. (2020). Comparison of indocyanine green and methylene blue use for axillary reverse mapping during axillary lymph node dissection. MedComm.

[44] Han J.W., Seo Y.J., Choi J.E., Kang S.H., Bae Y.K., Lee S.J. (2012). The efficacy of arm node preserving surgery using axillary reverse mapping for preventing lymphedema in patients with breast cancer. J. Breast Cancer.

[45] Noguchi M., Inokuchi M., Morioka E., Haba Y., Shioya A., Yamada S., Iida Y. (2024). Intraoperative nodal palpation is a mandatory component of sentinel lymph node biopsy for breast cancer. Arch. Breast Cancer.

[46] Tummel E., Ochoa D., Korourian S., Betzold R., Adkins L., McCarthy M., Hung S., Kalkwarf K., Gallagher K., Lee J.Y. (2017). Does axillary reverse mapping prevent lymphedema after lymphadenectomy?. Ann. Surg..

[47] Koshima I., Inagawa K., Urushibara K., Moriguchi T. (2000). Supermicrosurgical lymphaticovenular anastomosis for the treatment of lymphedema in the upper extremities. J. Reconstr. Microsurg..

[48] Colleoni M., Sun Z., Price K.N., Karlsson P., Forbes J.F., Thurlimann B., Gianni L., Castiglione M., Gelber R.D., Coates A.S. (2016). Annual hazard rates of recurrence for breast cancer during 24 years of follow-up: Results from the International Breast Cancer Study Group Trials I to V. J. Clin. Oncol..

[49] Pan H., Gray R., Braybrooke J., Davies C., Taylor C., McGale P., Peto R. (2017). 20-year risks of breast-cancer recurrence after stopping endocrine therapy at 5 years. N. Engl. J. Med..

